# Single-photon emission from colloidal perovskite nanocrystals

**DOI:** 10.1093/nsr/nwaf135

**Published:** 2025-04-17

**Authors:** Qingsen Zeng, Shinyoung Hwang, Hyeree Kim, Tae-Woo Lee

**Affiliations:** Department of Materials Science and Engineering, Seoul National University, Republic of Korea; Research Institute of Advanced Materials, Seoul National University, Republic of Korea; Department of Materials Science and Engineering, Seoul National University, Republic of Korea; Department of Materials Science and Engineering, Seoul National University, Republic of Korea; Department of Materials Science and Engineering, Seoul National University, Republic of Korea; Interdisciplinary Program in Bioengineering, Seoul National University, Republic of Korea; SN Display Co. Ltd, Republic of Korea; Institute of Engineering Research, Soft Foundry, Research Institute of Advanced Materials, Seoul National University, Republic of Korea

## Abstract

Colloidal perovskite nanocrystals emerge as promising single-photon sources, offering bright, coherent emission and room-temperature operation for future scalable quantum communication technologies.

A single-photon source (SPS) is a device that emits individual photons sequentially. SPSs are fundamental to the progress of quantum information technologies, in which photons convey quantum information. Photons have properties such as polarization, and spatial and temporal modes, which can be used to encode quantum information. This possibility can be exploited to increase the efficiency of quantum algorithms and for applications such as quantum-secure communication networks.

Currently, epitaxial self-assembled III–V quantum dots (QDs) are most widely used for quasi-deterministic single-photon emission [1]. They have set records in brightness, purity and indistinguishability. For example, epitaxial InAs QDs that are embedded in 1D optical microcavities have demonstrated a brightness of 56% at the detectors, purity of 95.9% and indistinguishability of 97.9% [2], which exceed those of alternatives [1] such as Er ions, color centers in diamonds, transition-metal dichalcogenides and defects in hexagonal boron nitride. These remarkable performances result from the tailored band structure, quantum confinement effect, reduced non-radiative recombination and defects, long coherence times and strong light–matter interaction in QD materials [3]. Under cryogenic conditions, these QDs deliver SPSs with high efficiency and consistency, and are therefore well suited for quantum information processing and communication.

However, brightness, purity and indistinguishability are not the only requirements. An ideal SPS must operate at room temperature, emit in the telecommunication band, be electrically driven and be easily compatible with scalable processing techniques. However, a source that has all these traits has not been achieved. Fabrication of state-of-the-art epitaxial III–V QDs requires expensive equipment that operates at high temperature and high vacuum. Furthermore, these QDs typically operate under cryogenic conditions.

Solution-processed colloidal QDs present a promising alternative by combining the unique advantages of QD materials with scalable, straightforward fabrication techniques. Among these, colloidal lead halide perovskite nanocrystals (PeNCs) stand out in single-photon research. Their broadly tunable sizes (4–30 nm), diverse compositions and unique photophysical properties make them highly versatile candidates [1,3,4]. In recent years, PeNCs have emerged as formidable challengers to the established family of colloidal QDs. Their easy synthesis, along with excellent light absorption, emission and charge-transfer properties, has driven rapid advances in photonic and optoelectronic applications.

PeNCs have several unique properties that make them ideal candidates for SPSs. Notably, PeNCs have good defect tolerance so they typically have fewer surface traps than colloidal QDs that use CdSe or InP without the need for passivation by epitaxial shells. As a result, the blinking phenomenon, which is commonly associated with surface trapping, can be suppressed at both cryogenic (Fig. 1a) [5] and room (Fig. 1b) temperatures [4]. Therefore, PeNCs could be employed as efficient quantum emitters that are capable of working at room temperature. Recent studies demonstrate that quantum confinement in PeNCs enhances single-photon purity by suppressing biexciton emission through accelerated Auger recombination at room temperature [6]. This effect has been systematically shown in CsPbBr_3_ and CsPbI_3_ PeNCs, in which smaller PeNC sizes exhibit a higher single-photon purity of 98% in strongly confined 6.6-nm CsPbI_3_ PeNCs. Additionally, phonon-driven wave-function localization has been demonstrated in organic–inorganic hybrid perovskite PeNCs such as FAPbBr_3_ (FA = formamidinium), introducing an alternative confinement mechanism [7]. Enhanced electron–phonon coupling in FAPbBr_3_ results in stronger excitonic quantum confinement than that in inorganic CsPbBr_3_, even for particles of similar size. This coupling drives partial localization of the excitonic wave function through polaron formation. Polaron stabilization increases charge carrier lifetimes by reducing Coulomb interactions and dielectric screening. As a result, the overlap between electron and hole wave functions decreases, extending exciton recombination lifetimes. Simultaneously, weaker Coulomb interactions lower the biexciton binding energy, which accelerates Auger recombination and shortens biexciton lifetimes [4]. This interplay allows large FAPbBr_3_ PeNCs to achieve a single-photon purity of 95% and stable, nearly blinking-free emission at room temperature. This strategy mitigates the necessity for extreme size reduction to enhance quantum confinement, which is often associated with reduced photostability.

**Figure 1.**
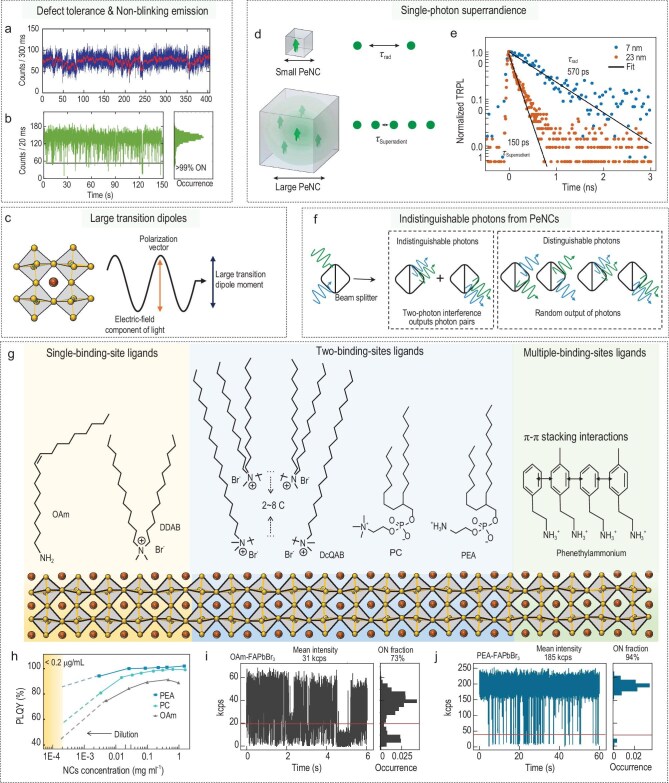
Unique photophysical properties and stability issues of PeNCs as single-photon emitters. (a, b) Photoluminescence (PL) intensity trajectories of single PeNCs: (a) CsPbBr_3_ at 3.6 K [5] and (b) FAPbBr_3_ at room temperature. (c) Schematic illustration showing optimal alignment of the oscillating electric field component of light (polarization vector) relative to the orientation of the transition dipole moment in perovskite. (d) Giant dipole formation, typically geometrically constrained in small PeNCs, is attainable as the PeNC size is increased and accelerates radiative decay (lifetime *τ*_Superradiant_) due to the increased exciton center-of-mass coherent volume. (e) Transient photoluminescence decay profiles of two single PeNCs—one lacking superradiance (lifetime *τ*_rad_, QD size 7 nm) and the other displaying superradiance (QD size 23 nm)—are shown for comparison [9]. (f) Photon indistinguishability in single PeNC is assessed through two-photon interference. When two indistinguishable photons reach the beam splitter simultaneously, they interfere and exit along the same optical path as a photon pair whereas distinguishable photons do not. (g) Chemical structures of ligands with single binding sites (OAm and DDAB), ligands with two binding sites (DcQAB, typical PC and typical PEA ligands) and supramolecular ligands with multiple binding sites (phenethylammonium and *N*-methylphenethylammonium). (h) Photoluminescence QY vs dilution for FAPbBr₃ PeNCs stabilized by OAm, PC or PEA [11]. Solid dots: experimental data; dashed lines project trends towards concentrations of 0.2 μg/mL, which correspond to the deposition of individual particles. (i) Single OAm-FAPbBr₃ PeNCs show pronounced blinking whereas (j) single PEA–PeNCs demonstrate minimal PL blinking and high brightness. Adapted from [4,5,9,11] with permission.

Furthermore, despite their smaller size (∼10 nm) compared with typical epitaxial QDs, PeNCs maintain a similarly large transition dipole moment (tens of Debyes, Fig. 1c), which indicates strong light–matter interactions [3]. Consequently, CsPbBr_3_ PeNCs have absorption cross sections that are two orders of magnitude larger than those of CdSe QDs, which emit at similar wavelengths to CsPbBr_3_ PeNCs [8]. Additionally, the large transition dipoles in PeNCs promote single-photon superradiance. A key distinction between PeNCs and classic CdSe-based QDs lies in their lowest band-edge fine energy levels. In PeNCs, the lowest energy state is a bright exciton state whereas, in CdSe-based QDs, it is typically a dark exciton state [3,5]. This difference fundamentally alters their emission dynamics. Conventional CdSe-based QDs exhibit slow, microsecond-scale emission at cryogenic temperatures due to relaxation from bright excitons to dark excitons. In contrast, PeNCs demonstrate an increase in the emission rate as the temperature decreases, as the delocalized exciton wave function enhances the transition dipole moment. This results in radiative-decay times of <100 ps at cryogenic temperatures for weakly confined PeNCs [9]. Consequently, the large transition dipole moments of PeNCs significantly increase the oscillator strength and accelerate radiative-decay rates (Fig. 1d and e). This collective behavior leads to single-photon superradiance, advancing the realization of bright and coherent SPSs.

Another remarkable feature is that the optical coherence time of PeNCs is comparable to their radiative-decay rate [5,10]. This alignment supports coherent single-photon emission and facilitates Hong–Ou–Mandel (HOM) two-photon interference. Another remarkable feature is the optical coherence time of the radiative-decay rate of PeNCs; this relationship enables coherent single-photon emission and HOM two-photon interference. Although the measured indistinguishability value of PeNC is 56% (Fig. 1f) [10], which is inferior to the best epitaxial QDs (>97%) [2], this is the first demonstration of such good indistinguishability in solution-processed colloidal QDs. This achievement is a significant step toward the development of scalable quantum emitters that can produce indistinguishable single photons.

In summary, PeNCs exhibit a unique combination of high defect tolerance and efficient quantum confinement effects, enabling high single-photon purity, non-blinking emission and room-temperature operation. Their large transition dipole moments and superradiant emission further enhance brightness and optical coherence. These combined properties position PeNCs as promising candidates for scalable quantum photonic applications.

However, despite these optical advantages, their structural stability remains a major challenge due to the fragile ionic lattice, which results in weak and dynamic binding of common ligands (e.g. oleylamine (OAm) in Fig. 1g) to the surface. During necessary dilution processes to deposit individual particles, ligand detachment leads to surface defects, significantly reducing the photoluminescence quantum yield (QY, Fig. 1h) and causing pronounced blinking and low brightness (Fig. 1i). Addressing these stability challenges requires the design of ligands with higher binding energy and multiple binding sites [11].

Quaternary ammonium bromides (e.g. DDAB) offer improved stability over OAm but are constrained by their single binding site [12]. In contrast, dicationic quaternary ammonium bromide (DcQAB) ligands, with two chelating binding sites, deliver superior surface stabilization for CsPbBr_3_ PeNCs. These ligands enable sub-200-ps lifetimes and coherent photon emission, even at cryogenic temperatures [13]. Similarly, long-chain zwitterionic ligands such as soy lecithin—an abundant, naturally derived phosphocholine (PC) surfactant—exhibit strong binding to PeNC surfaces. This enhances colloidal stability and preserves structural integrity in highly diluted solutions [14]. However, such ligands are less effective for hybrid PeNCs such as FAPbBr_3_ due to their softer lattice structure and potential ionic displacement equilibria, which can reduce QY upon dilution (Fig. 1g and h) [11].

Advancements in molecular engineering, such as primary-ammonium phosphoethanolamine (PEA) [11], enable the creation of lattice-matched zwitterionic surfactants. These ligands enhance both the structural and colloidal stability of hybrid organic–inorganic and lead-free perovskites, achieving a photoluminescence QY of >96% in solution and solid states, minimal intermittency and high single-photon purity (∼95%) with ON fractions of ≤94% (Fig. 1j). Such tailored surface chemistries represent a promising direction for stabilizing single PeNCs and advancing their application in quantum emitters.

However, the QY of PEA–PeNCs decreases upon dilution (Fig. 1h), suggesting that the two-binding-site PEA ligands still detach, resulting in a dynamic binding state on soft-lattice PeNCs. The development of ligands with stronger binding affinities and enhanced passivation capabilities remains a significant challenge. Polymer ligands with multiple binding sites show great potential for stabilizing single PeNCs. Recent studies reveal that π–π stacking interactions between phenethylammonium ligands induce the formation of supramolecular polymeric ligands (Fig. 1g), which significantly reduce surface defects and yield nearly non-blinking CsPbBr_3_ QDs with exceptional photostability and single-photon purity (∼98%) [15].

The use of epitaxial core–shell heterostructures offers another promising approach to stabilizing single PeNCs. The growth of a covalently bonded CdS shell on a CsPbBr_3_ core significantly improves the photostability of individual CsPbBr_3_ PeNCs and completely suppresses blinking [16]. However, the high-temperature epitaxial growth of a covalent-bond shell that is required for traditional core–shell QDs is incompatible with the soft lattice of PeNCs, and therefore leads to fusion of PeNC cores and failure to control the size and shell thickness. Therefore, the development of PeNC/metal chalcogenides with uniform shell thickness and controlled morphology remains a significant challenge. The growth of stable, wide-band-gap ionic crystal shells presents a promising solution.

Although PeNCs are strong candidates for SPS materials, several challenges must be addressed before they can be effectively applied in quantum information processing. To realize PeNCs as efficient SPSs, photon coherence must be increased and the fidelity of two-photon interference must be improved; these results can be achieved by minimizing single-particle spectral diffusion. This goal might be achieved by advances in synthetic chemistry and surface engineering. For instance, stable colloidal NC structures, achieved by surface ligands or core–shell configuration, can significantly reduce electron–phonon coupling in PeNCs. This reduction is crucial for narrowing the emission linewidth and enhancing photon coherence.

Research on perovskite SPSs has primarily focused on blue and green emitters (<540 nm) because they use perovskites that include Cl^–^ or Br^–^ and are more stable than perovskites that include I^–^. However, the red and near-infrared (NIR) range (∼650–790 nm) is critical for free-space quantum communication. Improvement of such emitters has been limited due to the instability of perovskites that include I^–^; they are susceptible to photo-oxidation, photobleaching and phase separation in Br^–^/I^–^ mixed halides [17,18]. Furthermore, most optical cavities are optimized for red and NIR wavelengths, but the limited number of studies on PeNC–cavity coupling have not yielded significant improvements in SPS performance [19,20]. Efforts should focus on the integration of single PeNCs into optical cavities to increase the optical coherence of emitted photons and achieve HOM interference visibility that is comparable to that of cutting-edge epitaxial QDs.

Coherent spin manipulation at room temperature would make it possible to perform quantum information processing without the need for cooling; this manipulation has not yet been demonstrated in epitaxial QDs, but has been achieved in ensemble PeNCs. This success is largely due to their strong spin–orbit coupling (from heavy elements such as Pb), efficient light–matter interaction and long spin coherence times. However, to build a universal set of quantum logic gates, single-particle spin manipulation is essential, as required by DiVincenzo's criteria [3]. This requirement underscores the need for cavity integration to strengthen light–matter interaction in single-photon emission.

Electrically driven PeNC single-photon emitters have not yet been realized because ionic PeNCs are unstable under electric fields. Ions within the PeNC lattice move when subjected to an electric field and this response destabilizes the material; consequently, the realization of such is a significant challenge. Ligand engineering and core–shell structuring aimed at suppressing ion migration are key strategies to overcome this challenge and enable the development of electrically driven PeNC SPSs.

Addressing these challenges will require collaborative efforts across diverse disciplines, including colloidal chemistry, semiconductor physics, nanofabrication and quantum engineering. We hope that this brief Perspective has provided a concise overview of the development, advantages and challenges of PeNC SPSs, and will stimulate collective efforts of chemists, materials scientists and device physicists to achieve low-cost, easily processed PeNC materials and increase the efficiency of single-photon emission.
